# Molecular and biophysical foundations of nano-enabled seed priming and smart nanocarriers for crop stress alleviation

**DOI:** 10.3389/fpls.2026.1954570

**Published:** 2026-09-18

**Authors:** Abdulbaki Shehu Abdulbaki, Hameed Alsamadany, Bolaji Umar Olayinka, Yahya Alzahrani

**Affiliations:** 1 Department of Plant Science, Faculty of Natural and Applied Sciences, Federal University of Agriculture and Development Studies Iragbiji, Iragbiji, Osun State, Nigeria; 2 Department of Biological Sciences, Faculty of Science, King Abdulaziz University, Jeddah, Saudi Arabia; 3 Department of Plant Biology, Faculty of Life Sciences, University of Ilorin, Ilorin, Nigeria

**Keywords:** abiotic stress, crop resilience, nanoparticle uptake, nanopriming, plant nanobiotechnology, seed priming, smart nanocarriers, sustainable agriculture

## Abstract

Agricultural productivity is increasingly constrained by interacting abiotic stresses, yet translation of nano-enabled seed treatments from controlled experiments to field agriculture remains limited. Seed nanopriming can initiate physiological and molecular priming before radicle emergence, but outcomes depend strongly on nanomaterial identity, particle size and morphology, surface charge, colloidal stability, cargo chemistry, exposure regime, and crop genotype. This review critically examines mesoporous silica nanoparticles, chitosan-based systems, metal–organic frameworks, hybrid/core–shell platforms, and catalytic nanozymes, distinguishing true cargo-delivery carriers from catalytic functional nanomaterials. We integrate interfacial physics with seed-coat and cell-wall barriers, clarifying how zeta potential, surface charge density, hydrodynamic size, porosity, functionalization, and material transformation influence adhesion, uptake, release, and translocation. At the molecular level, we connect nanoparticle–surface interactions with ROS and Ca2+ signaling, membrane-potential changes, MAPK/CDPK cascades, phytohormone cross-talk, transcription-factor activation, antioxidant defenses, and stress-memory processes. We also distinguish transient physiological memory from mitotically persistent and experimentally demonstrated transgenerational inheritance, and critically examine bio–nano combinations in soil, including dissolution, redox transformation, heteroaggregation, and eco-corona formation. Particular emphasis is placed on dose- and material-dependent phytotoxicity, multi-stress cross-tolerance, regulatory status of transgene-free editing, and practical barriers involving reproducibility, cost, scalability, field stability, and environmental safety. Finally, we identify realistic research priorities, including standardized nanoformulation reporting, field-validated dose windows, multi-omics and mechanistic imaging, Nano-QSAR/graph-learning workflows, and safer-by-design criteria for precision seed treatment.

## 1 Introduction

Agricultural productivity is increasingly threatened by climate variability, soil degradation, water scarcity, and the increasing frequency of concurrent stress combinations. Although drought, salinity, heat, and metal toxicity are often studied separately, field-grown crops experience overlapping perturbations that converge on osmotic imbalance, ionic toxicity, oxidative stress, membrane dysfunction, and altered carbon allocation. Nano-enabled seed treatments are attractive because they can deliver a small chemical or biological stimulus before sowing and potentially establish a primed state without requiring continuous foliar or soil application. However, the literature does not support a universal ‘nanopriming effect’: responses vary with material chemistry, size, surface functionalization, dose, exposure duration, seed coat properties, genotype, and surrounding soil or solution chemistry (Singh et al., 2025; Atanda et al., 2025).

Seed priming is a controlled hydration process that activates metabolic repair and signaling while avoiding radicle protrusion. Nanopriming adds a second layer of control because the nanomaterial can act as a carrier, elicitor, nutrient source, redox catalyst, or combination of these functions. A recent meta-analysis of drought experiments across crops found that engineered nanomaterials increased relative water content by 10.8% and water-use efficiency by 33.3%, with seed application showing greater protective potential than root or foliar application; nevertheless, the analysis also identified dependence on crop type and nanomaterial characteristics (Aslam et al., 2023; Basu et al., 2021; Chen et al., 2025; Janah et al., 2025; Khalid et al., 2023).

The central knowledge gaps addressed in this review are fourfold. First, physicochemical descriptors are often reported separately from plant-response data, making it difficult to determine which material attributes actually control uptake and efficacy. Second, many mechanistic discussions stop at changes in ROS, hormones, or antioxidant enzymes without tracing the upstream signaling sequence from interfacial events to Ca2+/MAPK/CDPK signaling and transcriptional regulation. Third, evidence from hydroponic or laboratory systems is frequently extrapolated to soil and field environments without accounting for dissolution, aggregation, redox transformation, or eco-corona formation. Fourth, claims of hormesis, transgenerational memory, and environmental compatibility are sometimes broader than the underlying evidence permits. The review therefore emphasizes conditionality, comparative performance, mechanistic evidence strength, and translational feasibility.

## 2 Physical chemistry and structural orientation of advanced smart nanocarriers

The functional classes considered here are not equivalent. MSNs, chitosan nanoparticles, MOFs, and hybrid/core–shell systems are primarily engineered as carriers in which pore structure, polymer matrices, coordination chemistry, or compartmentalization govern cargo loading and release. Nanozymes such as CeO2 and Mn3O4 are better viewed as catalytic or biologically active nanomaterials: they may carry cargo when engineered as composites, but their defining agricultural function is redox catalysis or enzyme-mimetic activity. A useful comparison therefore considers loading capacity, colloidal stability, interfacial adhesion, cellular access, release mechanism, catalytic activity where applicable, agronomic evidence, and failure modes rather than treating all nanomaterials as interchangeable (**Table 1**).

**Table 1:** Comparative critical assessment of major nanomaterial classes considered for nano-enabled seed treatment.

Class	Primary function	Typical design attributes	Interfacial/uptake considerations	Release or catalytic mechanism	Key strengths	Principal limitations
MSNs	Cargo carrier	Mesoporous silica; tunable pore size; surface functionalization	Hydrodynamic size and surface charge govern adhesion and mobility; aggregation can close pores	Diffusion/pore gating; stimuli-responsive caps	High loading capacity; modular chemistry; protects unstable cargo	Complex synthesis; aggregation in ionic media; limited field validation
Chitosan nanoparticles	Carrier + elicitor	Biodegradable polymer; molecular weight and DD strongly influence charge	Positive charge favors seed/cell-wall adhesion but can increase retention at high charge	Swelling, diffusion, biodegradation, pH-dependent protonation	Biodegradability; intrinsic elicitation; scalable ionic gelation	Batch variability; viscosity/aggregation; dose-dependent phytotoxicity
ZIF-8 / Fe-MOFs	Cargo carrier + nutrient source	Porous coordination networks; metal/ligand tunability	Size, ligand chemistry and degradation govern exposure; matrix pH and redox matter	pH- or redox-responsive framework dissociation	High chemical tunability; triggered release	Cost and synthesis complexity; metal/ligand residues; soil transformation uncertainty
Hybrid/core–shell systems	Multifunctional carrier	Lipid/phenolic/metal/ceramic combinations	Multiple interfaces can improve protection but create complex corona and aggregation behavior	Sequential or multi-trigger release	Combines protection, targeting and co-delivery	Higher manufacturing complexity; reproducibility and scale-up barriers
CeO2 / MnOx nanozymes	Catalytic functional material	Redox-active oxide; defect and oxidation-state dependent	Charge and size affect localization; dissolution and corona can alter activity	SOD/catalase/peroxidase-like catalysis	Direct ROS regulation; catalytic rather than stoichiometric action	Apparent kinetics are assay-specific; excessive redox activity can be harmful; not primarily cargo carriers

### 2.1 Mesoporous silica nanoparticles

Mesoporous silica nanoparticles (MSNs) are among the most extensively investigated nanocarriers for agricultural applications because of their highly ordered porous architecture and exceptional loading capacity. Their three-dimensional silica framework contains interconnected pores ranging from approximately 2 to 50 nm in diameter, providing a large internal surface area capable of accommodating diverse agrochemicals (Wang et al., 2023). These nanoparticles are commonly synthesized through CTAB-templated sol-gel reactions using tetraethyl orthosilicate (TEOS) as the silica precursor. Subsequent surface modification with organosilanes such as (3-aminopropyl) triethoxysilane (APTES) introduces reactive amino groups that improve cargo binding and enable further functionalization (Lee et al., 2025).

The porous network of MSNs allows efficient encapsulation of both hydrophobic and hydrophilic molecules while protecting sensitive compounds against ultraviolet radiation, thermal degradation, and premature leaching after application (Wang et al., 2023). Controlled release can be achieved by sealing pore openings with stimuli-responsive polymers or molecular gatekeepers that respond to environmental or biological signals. Depending on the carrier design, cargo release generally follows zero-order, Higuchi, or Korsmeyer-Peppas kinetic models, enabling sustained and targeted delivery under field conditions (Lee et al., 2025).

For MSNs, pore diameter and surface chemistry jointly determine cargo loading and release. Larger accessible mesopores can accommodate bulky molecules, whereas aminosilanization or other functionalization changes electrostatic interactions with both cargo and plant surfaces. The principal advantage is architectural tunability; the principal uncertainty is whether a formulation characterized in buffer retains the same pore accessibility and colloidal size in the ionic, organic-rich environment encountered during imbibition or in soil. Thus, nominal particle diameter should not be interpreted as the sole predictor of uptake; hydrodynamic diameter, agglomeration state, surface corona, and seed-coat adsorption must be measured under application-relevant conditions.

### 2.2 Chitosan nanoparticles

Chitosan nanoparticles (CNPs) are biodegradable polymeric carriers and also function as elicitors. Their behavior depends strongly on molecular weight and degree of deacetylation (DD), which govern the density of protonatable amino groups, chain conformation, hydrodynamic size, and zeta potential. Commercial and research-grade chitosans commonly span broad ranges of approximately 50–1,000 kDa in molecular weight and 70–95% DD, and these ranges should be reported explicitly because nominally identical ‘chitosan nanoparticles’ can therefore have very different interfacial and biological properties. At acidic pH, protonation of amino groups increases positive charge and promotes binding to negatively charged seed and cell-wall surfaces; increasing DD generally increases the number of available amino groups, whereas molecular-weight effects can alter viscosity, aggregation, particle formation, and release kinetics. Consequently, chitosan formulation studies should report DD, molecular weight, pH, ionic strength, particle size by both microscopy and dispersion-based methods where possible, and zeta potential rather than relying on particle size alone.

Most CNPs are produced by ionic gelation, commonly using sodium tripolyphosphate (TPP) as the crosslinker. The same positive surface charge that improves seed adhesion can become a limitation when excessive: strong adsorption may increase surface retention, alter mucilage formation, and reduce mobility. Accordingly, chitosan should be considered a tunable interface rather than intrinsically ‘safe’ or ‘superior’; its agronomic performance is dose- and formulation-dependent (Pereira et al., 2021; Soni et al., 2023).

Recent advances have expanded the functionality of chitosan-based systems through the development of nanocomposites incorporating zinc oxide nanoparticles within CS/TPP matrices. Green synthesis methods employing plant extracts, including *Helichrysum odoratissimum*, facilitate the formation of ZnO nanoparticles directly inside the chitosan framework, yielding particles approximately 149–257 nm in diameter with strong positive surface charges (Zungu et al., 2023). These nanocomposites exhibit excellent adhesion to seed surfaces and significantly improve early seedling establishment under saline conditions. Similarly, encapsulation of highly diluted garlic extract (<0.1%) within chitosan nanoparticles produces stable spherical nanostructures of approximately 200 nm that function as environmentally friendly biostimulants capable of markedly enhancing the expression of growth- and defense-associated genes in cereal crops (Mondéjar-López et al., 2024).

The biological response to chitosan is strongly concentration dependent. At relatively low concentrations, chitosan enhances water uptake and stimulates germination. However, excessive concentrations increase solution viscosity and may form a dense coating over the seed surface, restricting oxygen diffusion into the embryo and ultimately triggering programmed cell death (Pereira et al., 2021).

### 2.3 Metal-organic frameworks

Metal–organic frameworks (MOFs) constitute a distinct class of crystalline porous materials assembled from inorganic metal ions or clusters interconnected by multifunctional organic ligands (Li et al., 2024). Their exceptionally high porosity, structural tunability, and stimulus-responsive behavior make them highly attractive nanocarriers for precision agriculture.

Among these materials, Zeolitic Imidazolate Framework-8 (ZIF-8), synthesized from zinc ions and 2-methylimidazole, has emerged as one of the most widely investigated systems for controlled agrochemical delivery (Chen et al., 2024). The coordination bonds that stabilize the framework remain intact under neutral conditions but readily dissociate in mildly acidic environments (pH 5.0–6.0). Protonation of the imidazole ligands destabilizes the structure, resulting in the controlled release of encapsulated pesticides, fertilizers, or plant growth regulators precisely where they are required (Chen et al., 2024). As the framework degrades, bioavailable zinc ions are simultaneously released, supplying an essential micronutrient that contributes to chlorophyll synthesis and normal plant growth.

Iron-based MOFs, including MIL-101(Fe) and NH_2_-MIL-101(Fe), display a complementary mode of responsiveness. These materials undergo degradation in response to intracellular redox conditions, such as elevated glutathione concentrations, or within pathogen-induced acidic microenvironments, thereby releasing both encapsulated agrochemicals and nutritionally important iron ions to target tissues (Li et al., 2024).

### 2.4 Advanced hybrid and core-shell platforms

To integrate multiple biological functions within a single delivery platform, researchers have increasingly developed hybrid nanostructures and core–shell architectures that combine controlled release with additional protective properties.

One example is the Phelm platform, a multicomponent nanocarrier consisting of a hydrophobic lipid core enclosed within a tannic acid–iron (Fe³^+^) coordinated phenolic shell (Aguilar Perez et al., 2026). The lipid core serves as a reservoir for plant growth regulators such as indole-3-acetic acid, whereas the surrounding phenolic shell regulates their gradual release. In addition to controlling hormone delivery, the shell scavenges excessive reactive oxygen species and provides bioavailable iron, thereby simultaneously supporting growth and protecting plants exposed to salinity stress (Aguilar Perez et al., 2026).

Another notable innovation is the metal–phenolic nanocarrier CAFZin-K, which is produced through the coordination-driven self-assembly of zinc ions and caffeic acid (Aguilar Perez et al., 2026). This platform encapsulates kinetin and increases its cellular uptake by approximately 3.7-fold relative to the free hormone. Its controlled, stress-responsive release minimizes premature losses while maintaining hormone availability during prolonged drought and salinity stress (Aguilar Perez et al., 2026).

Hybrid composites have also been developed by coating ZIF-8 nanoparticles with hydroxyapatite (HA). The resulting ZIF-8@HA structures exhibit markedly slower degradation in aqueous environments, extending structural stability from approximately four hours to nearly seven days (168 hours) (Sharma et al., 2023). This prolonged stability enables sustained release of nitrogen, phosphorus, and potassium while simultaneously providing antimicrobial activity against plant pathogens such as *Pseudomonas syringae* (Sharma et al., 2023).

### 2.5 Catalytic nanozymes as functional nanomaterials

Nanozymes are included here as a separate functional category because their principal contribution is catalytic control of reactive oxygen species (ROS), not conventional cargo delivery. Cerium oxide (CeO2) exploits Ce3+/Ce4+ redox cycling and oxygen vacancies, while manganese oxide materials can display SOD-, catalase-, and peroxidase-like activities. These materials can be incorporated into carriers or coatings, but their catalytic activity is mechanistically distinct from pore-based or polymer-based cargo release (Wu et al., 2024; Wang et al., 2025).

Quantitative kinetic data are highly formulation-specific and are generally obtained in cell-free assays rather than in seeds. For example, a porous CeO2 nanozyme has been reported to show catalase-like activity with a Km of 4.69 mM H2O2 and a Vmax of 11.7 × 10−7 M s−1 in the dark, with activity changing substantially under visible-light irradiation. Such values should not be treated as direct equivalents of native catalase because surface area, defect density, illumination, assay composition, and particle concentration affect apparent kinetics. Likewise, published Mn-containing nanozyme studies report catalytic parameters that vary markedly with composition and assay design. Where agricultural studies do not provide Michaelis–Menten parameters, it is more rigorous to report catalytic activity relative to a defined assay endpoint than to imply enzyme equivalence (Li et al., 2025).

In seed-priming applications, nanozymes may therefore be most useful when a calibrated catalytic window is combined with favorable interfacial behavior. At excessive doses, the same redox activity that supports H2O2 regulation can become disruptive if ROS generation, dissolution products, or membrane interactions exceed the plant’s buffering capacity. Biogenic MnO nanoparticles likewise require material-specific characterization because biological synthesis can leave adsorbed proteins, metabolites, or capping agents that alter surface reactivity and toxicity; ‘green’ synthesis alone does not establish environmental safety.

Biogenic metal-oxide nanozymes remain promising because microbial or plant-mediated synthesis can alter nucleation, defect density, surface ligands, and aggregation. However, environmentally favorable synthesis routes should be evaluated with the same rigor as conventional routes, including residual biomolecules, batch-to-batch reproducibility, dissolution behavior, particle-size distributions, surface chemistry, and dose–response relationships.

## 3 Zeta potential, surface charge dynamics, and interfacial interactions at biological barriers

The behavior of nanoparticles within plant systems is governed not only by their chemical composition but also by their interfacial properties. Among these, surface charge, particle size, and colloidal stability play decisive roles in determining nanoparticle adhesion, mobility, cellular uptake, and long-distance transport. Understanding these physicochemical interactions is therefore essential for designing nanocarriers capable of efficiently crossing biological barriers while minimizing aggregation and off-target deposition.

Dynamic Light Scattering (DLS) is routinely employed to determine the hydrodynamic diameter of nanoparticles suspended in solution. Unlike measurements of the solid particle alone, DLS accounts for the hydration layer and adsorbed surface molecules that surround each nanoparticle, thereby providing an estimate of its effective size under physiological conditions (Zhang et al., 2023). In contrast, Transmission Electron Microscopy (TEM) measures the dry core diameter after solvent removal, which typically results in smaller particle-size estimates because hydration shells and surface coronas are absent during imaging.

Surface charge density, Stern potential, and zeta potential are related but non-identical descriptors. Surface charge density describes the amount of electric charge per unit area at the material interface; the Stern potential refers to the electrical potential near the boundary between specifically adsorbed ions and the diffuse layer; zeta potential is an electrokinetic potential estimated near the shear plane during particle motion in a liquid. Accordingly, zeta potential is a practical indicator of colloidal stability and electrostatic interaction tendency, but it should not be interpreted as a direct measurement of true surface charge density or the exact potential at the solid surface. This distinction matters in plant systems because adsorbed pectins, salts, proteins, mucilage, and other solutes can shift the shear-plane environment during seed imbibition.

Positive zeta potential can increase adhesion to negatively charged pectin-rich surfaces, whereas a more negative or near-neutral zeta potential may reduce initial adsorption and increase mobility. However, neither polarity is universally optimal: strong positive charge may increase retention and aggregation at the seed surface, while ionic-strength-induced double-layer compression can reduce the magnitude of zeta potential and promote agglomeration. Therefore, reported values should be interpreted together with hydrodynamic diameter, polydispersity, ionic strength, pH, and the measurement medium rather than treated as transferable constants.

Chitosan-based nanoparticles, typically exhibiting zeta potentials around +45.3 mV, and zinc-loaded chitosan nanocomposites with surface charges ranging from +35 to +45 mV, demonstrate particularly strong affinity for negatively charged cell walls because of these ionic interactions (Zungu et al., 2023). Likewise, positively charged carbon dots (+24 to +34 mV) exhibit pronounced binding to pectin-rich tissues (Zhang et al., 2023). Although enhanced adhesion can improve localized delivery, excessively high positive surface charges may stimulate root mucilage secretion, resulting in nanoparticle entrapment at the root surface and reduced internalization.

Nanoparticles possessing negative surface charges exhibit markedly different transport behavior. Polyacrylic acid-coated cerium oxide nanoparticles, with zeta potentials ranging between −25 and −35 mV, experience electrostatic repulsion from negatively charged plant cell walls, thereby reducing surface adsorption (Wu et al., 2018). Rather than limiting delivery, this reduced adhesion facilitates greater mobility through the apoplastic continuum, allowing negatively charged nanoparticles to migrate more efficiently within extracellular spaces. Neutral nanoparticles, typically exhibiting zeta potentials between 0 and −5 mV, interact only weakly with plant surfaces and therefore depend largely on passive diffusion, making successful uptake highly dependent on maintaining sufficiently small hydrodynamic diameters.

The colloidal stability of nanoparticle suspensions is not fixed but is strongly influenced by environmental conditions. Increasing nanoparticle concentration or elevating the ionic strength of the surrounding medium compresses the electrical double layer surrounding each particle, thereby reducing the magnitude of the zeta potential. As electrostatic repulsion weakens, aggregation becomes increasingly likely, resulting in larger particle clusters with diminished mobility during seed imbibition.

To counteract this limitation, nanoparticles are frequently functionalized with organic stabilizing ligands such as citrate. These surface coatings provide electrosteric stabilization by combining steric hindrance with electrostatic repulsion, thereby maintaining particle dispersion even under saline or high-ionic-strength conditions. Such stabilization is particularly important during seed priming, where sustained nanoparticle mobility is necessary to facilitate penetration into embryonic tissues and ensure efficient delivery of bioactive cargo.

## 4 Biophysical and anatomical barriers to nanoparticle uptake and translocation

Following successful adhesion to the seed surface, nanoparticles encounter a succession of anatomical and physiological barriers that regulate their movement into plant tissues (**Figure 1**). The efficiency of nanopriming therefore depends not only on nanoparticle design but also on the ability to overcome these structural constraints while preserving cellular integrity.

**Figure 1:**
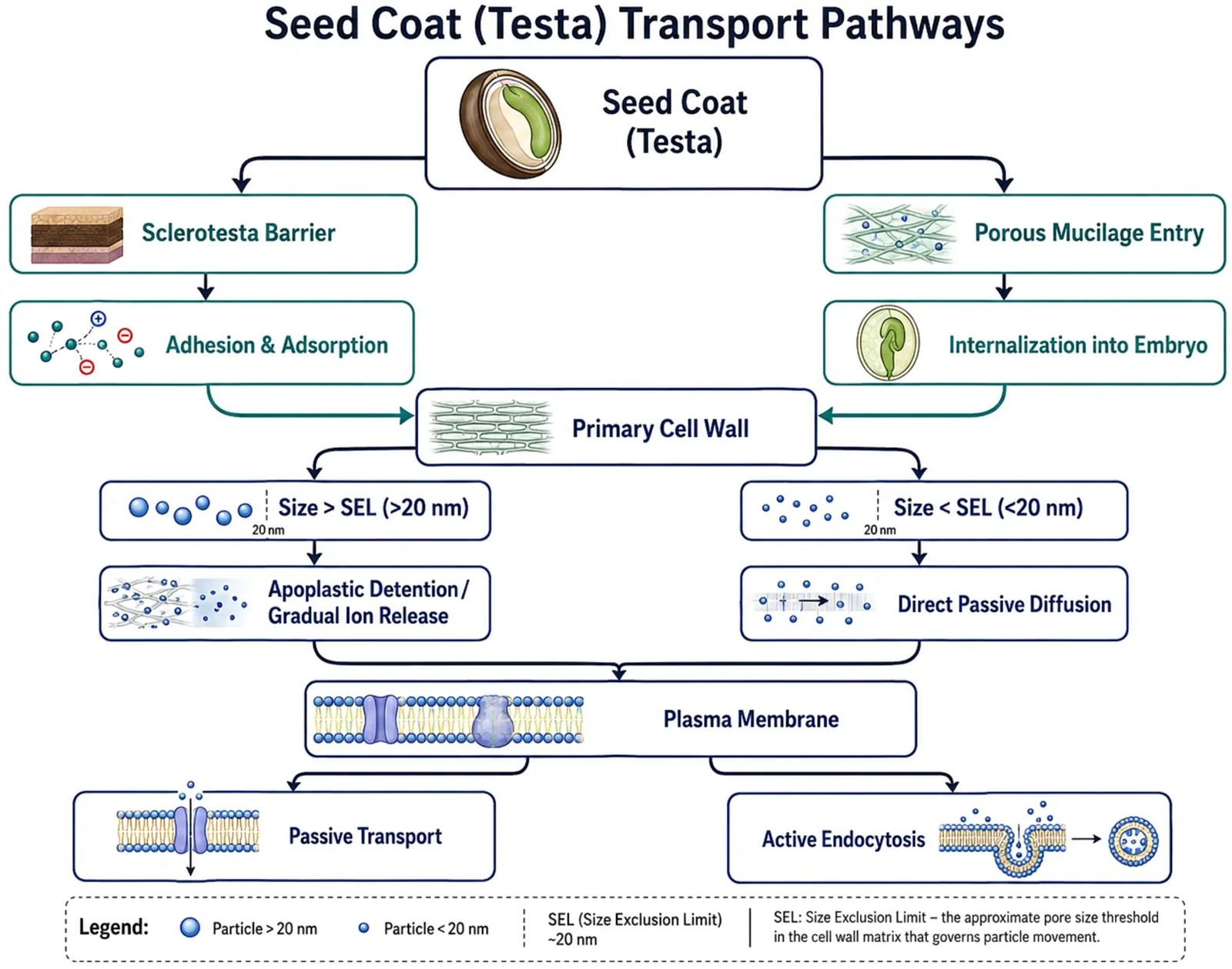
Seed coat transport pathways governing nanoparticle entry into the embryo.

The plant cell-wall size-exclusion limit (SEL) should be treated as an approximate, tissue- and condition-dependent descriptor rather than a universal pore diameter. Values in the broad range of approximately 5–20 nm have been reported for model and native cell-wall systems, but effective porosity varies with tissue type, developmental stage, hydration state, cellulose–hemicellulose–pectin composition, wall remodeling, and particle surface chemistry. Moreover, nanoparticle hydrodynamic diameter can differ substantially from dry TEM diameter because of hydration layers, adsorbed ions, protein or organic coronas, and aggregation. Thus, a particle larger than the nominal SEL may still be detected beyond the wall through deformability, wall remodeling, transient defects, endocytic routes, or other non-size-exclusive pathways, whereas a nominally small particle can remain immobilized by surface interactions (Demirer et al., 2021; Zhang et al., 2023).

A variety of analytical techniques have been employed to investigate nanoparticle internalization within plant tissues. Neutron activation analysis, transmission electron microscopy (TEM), and gas chromatography–mass spectrometry (GC–MS) have collectively demonstrated that both hydrophobic core–shell nanoemulsions and silver nanoparticles can successfully penetrate the outer testa and accumulate within embryonic tissues, including the cotyledons (Demirer et al., 2021).

Not all nanomaterials rely solely on passive diffusion through existing cell wall pores. High-aspect-ratio nanostructures, particularly single-walled carbon nanotubes (SWCNTs) functionalized with Pluronic polymers (P85-SWCNTs), are capable of entering plant cells via the Lipid Exchange Envelope Penetration (LEEP) mechanism (Krumova et al., 2023). This pathway enables direct access to the cytoplasm and chloroplasts without causing detectable disruption of either the cell wall or plasma membrane, highlighting the importance of nanoparticle geometry in determining cellular uptake.

Once internalized, nanoparticles are transported throughout the plant via two principal pathways:

- The apoplastic route is best viewed as extracellular movement through cell walls and intercellular spaces, constrained by adsorption, tortuosity, aggregation, and the Casparian strip. Particle size and morphology determine steric accessibility; surface charge and functionalization determine adsorption versus dispersion; and hydrophobicity or amphiphilicity can alter membrane and lipid interactions. The symplastic route requires passage across the plasma membrane or another cellular entry mechanism and subsequent movement through plasmodesmata or the vascular system. Evidence for xylem and phloem transport exists for selected nanomaterials and experimental systems, but long-distance movement is highly material- and plant-dependent and should not be generalized from detection in one tissue to systemic delivery in all crops.
- The symplastic pathway involves entry into the cytoplasm and movement between adjacent cells connected by plasmodesmata. Once nanomaterials or their released ions/cargo enter the vascular stream, xylem transport is generally associated with acropetal movement and phloem with source-to-sink redistribution. Direct evidence for intact nanoparticle movement through both streams is more limited than evidence for ion or molecular cargo movement; therefore, claims of systemic nanoparticle transport should specify whether the measured species is the intact particle, a dissolved constituent, or a released cargo.

Overall, nanoparticle transport is a coupled process in which size, shape, deformability, surface chemistry, corona formation, dissolution, and plant anatomy jointly determine localization. This is particularly important for seed nanopriming because the first transport step occurs during imbibition, before root and vascular barriers are fully established.

## 5 Molecular networks, transcriptional regulation, and epigenetic stress memory

Nano-enabled seed priming exerts its benefits through a coordinated reprogramming of molecular, biochemical, and physiological processes rather than by simply improving water uptake during germination. Upon exposure to nanoparticles, seeds undergo extensive transcriptional, metabolic, and signaling adjustments that collectively enhance their capacity to withstand subsequent abiotic stresses. These molecular changes influence hormone homeostasis, redox regulation, gene expression, and epigenetic programming, ultimately establishing a physiological state that enables faster and more effective stress adaptation.

### 5.1 Modulation of phytohormone signaling and ROS homeostasis

A more mechanistic model begins at the nano–bio interface. Adsorption of nanoparticles or released ions to seed-coat and plasma-membrane surfaces can alter membrane electrostatics, ion-channel activity, redox state, or local ROS production. These early perturbations can generate transient Ca2+ signatures and membrane depolarization or repolarization, followed by activation of calcium sensors such as calmodulin/Ca2+-dependent protein kinases and mitogen-activated protein kinase (MAPK) cascades. MAPK and CDPK signaling can then phosphorylate stress-responsive transcriptional regulators, including WRKY, MYB, NAC and AP2/ERF-family factors, while ROS such as H2O2 act as local and mobile second messengers. The resulting transcriptional response interfaces with ABA, salicylic acid (SA), jasmonic acid (JA), auxin, and gibberellin signaling, altering stomatal behavior, antioxidant metabolism, ion transport, osmolyte accumulation, and growth. The key point is that nanopriming should be understood as a perturbation of interconnected signaling networks, not as a direct one-step increase in antioxidant enzyme activity.

CeO2 nanoparticle priming illustrates one possible downstream branch: altered redox status is associated with changes in ABA/GA/JA balance and enhanced starch mobilization during germination. ZnO, TiO2, SiNPs and AgNPs can engage different branches depending on dissolution, surface chemistry, dose and tissue exposure. For example, Zn availability may alter antioxidant and ion-homeostasis networks, whereas catalytic CeO2 can directly modify ROS turnover. These examples should not be collapsed into a single mechanism because the same endpoint—improved stress tolerance—can arise through distinct proximal signals.

Comparable hormonal adjustments have been reported for other nanomaterials. Zinc oxide (ZnO) nanoparticle priming lowers endogenous ABA concentrations under drought conditions, whereas titanium dioxide (TiO_2_) nanoparticles preferentially stimulate auxin (IAA) accumulation in aerial tissues. This spatial differentiation in hormone regulation promotes coordinated root growth and shoot development, thereby improving whole-plant vigor under water-deficit conditions (Abou-Zeid et al., 2021; Rehman et al., 2025; Ye et al., 2026).

ROS signaling should also be distinguished from oxidative injury. At an appropriate exposure level, a transient H2O2/ROS increase can function as a signal that activates antioxidant, calcium, MAPK, and transcription-factor networks; at excessive exposure, persistent ROS can cause lipid peroxidation, protein oxidation and nucleic-acid damage. The dose therefore determines whether the material acts as a priming signal, a benign nutrient source, or a stressor that exceeds cellular buffering capacity (Chen et al., 2023; Azupio et al., 2026).

The resulting network can be summarized as: nanoparticle/cargo interaction → altered surface or intracellular redox state → Ca2+/membrane-potential signaling → CDPK/MAPK activation → transcription-factor phosphorylation and gene regulation → hormone/ion/antioxidant adjustment → altered physiology and stress resilience. Evidence for each step is stronger for plant stress signaling in general than for every nanomaterial formulation; consequently, direct nanopriming mechanistic claims should be matched to the evidence available for the specific material and crop.

Stress memory also requires careful terminology. Short-term transcriptional or metabolic memory refers to a primed physiological state that persists after the original stimulus, whereas mitotically persistent memory implies retention through cell divisions. Transgenerational inheritance requires demonstrated transmission through reproductive generations, ideally with molecular and phenotypic evidence in progeny. Current nanopriming literature provides stronger support for transient and within-generation priming than for stable, reproducible transgenerational inheritance. Therefore, nanopriming-associated epigenetic changes should be regarded as promising candidate mechanisms rather than assumed proof of inherited stress tolerance.

Potential epigenetic mechanisms include altered DNA methylation in CG/CHG/CHH contexts, with MET1 and DRM2 representing major maintenance and *de novo* methylation activities, respectively; remodeling of active H3K4me3 and repressive H3K27me3 marks at stress-responsive loci; and small-RNA/RdDM pathways that can alter transcriptional competence. These mechanisms are well established in plant stress memory generally, but they are not yet resolved as universal molecular signatures of seed nanopriming. Recent salt-priming work in Arabidopsis linked priming-associated shifts in H3K4me3/H3K27me3 with OPR3, AOC1 and LOX3 expression, illustrating the type of locus-specific evidence needed to claim chromatin-mediated memory. Accordingly, future nanopriming studies should combine whole-genome methylomes, ChIP-seq/CUT&Tag for histone marks, small-RNA sequencing, and generation-resolved phenotyping rather than infer transgenerational inheritance from a single-generation stress phenotype (Arıkan et al., 2026; Zaid et al., 2026).

### 5.2 Specific gene expression profiling

- Silicon Nanoparticles (SiNPs): Enhance photosynthetic performance by upregulating genes involved in chlorophyll biosynthesis, including *BnCAO*, *BnCHLG*, and *BnPOR*, while simultaneously suppressing the senescence-associated gene *BnSAG12*, thereby delaying leaf aging under stressful conditions (Ulhassan et al., 2025). SiNPs stimulate the expression of *BnPAL*, *BnCAD*, and *BnPPO*, genes that participate in the phenylpropanoid pathway and lignin biosynthesis.
- Zinc Oxide Nanoparticles (ZnO NPs): Exert broad regulatory effects on oxidative defense and ionic homeostasis. In barley exposed to salinity stress, ZnO nanopriming significantly increases expression of *CAT1*, *APX*, *SODA*, *GPX*, and *GR*, strengthening enzymatic detoxification of ROS (Abozaid et al., 2026). Simultaneously regulates ion transport by enhancing *NHX1*, *NHX2*, *NHX3*, *SOS1*, and *SOS3*.
- Garlic-Extract Chitosan Nanoparticles: RNA sequencing reveals widespread upregulation of genes involved in metabolism, cellular development, stress signaling, and growth regulation (Mondéjar-López et al., 2024).

## 6 Bio–nano treatment combinations and rhizosphere engineering

Combining nanomaterials with plant-growth-promoting rhizobacteria (PGPR) is promising, but the term ‘synergy’ should be reserved for experiments that demonstrate a statistical interaction between the components, typically through a factorial design. Where such interaction tests are absent, the safer terms are combination, co-application, or additive effect. This distinction is important because nanomaterials can either support microbial establishment or inhibit sensitive taxa through metal-ion release, oxidative stress, membrane interactions, or altered nutrient availability (Kareem et al., 2025; Campos et al., 2023).

In soil, the applied nanoparticle rarely remains in its pristine state. Particle dissolution, oxidation–reduction reactions, sulfidation where sulfur is available, homo- and heteroaggregation, sorption to minerals, and association with dissolved organic matter can change size, charge, reactivity, and bioavailability. These processes differ among calcareous, acidic, clay-rich, sandy, and organic-matter-rich soils. Natural organic matter and root exudates can form dynamic eco-coronas that modify colloidal stability, root attachment, microbial recognition, and toxicity. Recent work shows that the chemical composition of soil-derived organic matter can materially change corona structure, aggregation, and nanoparticle bioavailability (Fregolente et al., 2026; Leszczuk and Zając, 2026).

For PGPR, the practical implication is that a nominal nanoparticle dose does not necessarily equal the biologically available dose at the root surface. Eco-corona formation may reduce surface reactivity and toxicity, or in other contexts preserve mobility and facilitate plant or microbial exposure. Similarly, dissolution can convert a nanoparticle into an ionic nutrient or toxicant, so studies should measure both intact particles and dissolved fractions where relevant. Therefore, rhizosphere engineering should be evaluated with soil-specific measurements of dissolved metal, particle-size distribution, redox state, organic-matter binding, microbial abundance, and root colonization rather than relying on solution-phase characterization alone.

## 7 Nano-enabled light harvesting and photoprotection

Recent advances in plant nanobiotechnology have extended the application of nanomaterials beyond stress mitigation to include direct enhancement of photosynthetic performance. By interacting with chloroplasts and photosynthetic machinery, selected nanomaterials can improve light capture, optimize energy conversion, and protect photosystems from environmental damage.

Carbon nanotubes (CNTs) have emerged as one of the most extensively investigated nanomaterials for improving photosynthetic efficiency. Owing to their unique optical and electronic properties, CNTs can integrate into chloroplasts and expand their capacity to absorb a broader spectrum of incident light (Demirer et al., 2021). Seed priming with single-walled carbon nanotubes functionalized with Pluronic P85 (P85-SWCNTs) has likewise demonstrated significant physiological benefits, increasing the proportion of photochemically active Photosystem II (PSII) reaction centers in a concentration-dependent manner (Krumova et al., 2023).

### 7.1 Cross-tolerance under multifactorial stress

Because field environments rarely present a single stress, nanopriming should be evaluated against combined-stress scenarios such as drought plus heat or salinity plus heavy metals. Cross-tolerance may emerge when priming converges on shared nodes such as ROS/Ca2+ signaling, antioxidant capacity, ion transport, and hormone cross-talk. However, hormone interactions are not necessarily synergistic: ABA is often central to osmotic and drought responses, whereas SA and JA can promote defense programs that compete for carbon and signaling capacity. A nanopriming treatment that improves one component of a combined stress could therefore worsen another if it shifts the ABA–SA–JA balance beyond an optimal range. Mechanistic studies should measure hormone pools, key biosynthetic/signaling genes, ion ratios, photosynthetic performance, and yield under factorial multi-stress designs rather than infer broad cross-tolerance from single-stress experiments.

## 8 Phytotoxicity, concentration thresholds, and the safer-by-design paradigm

A dose–response relationship is central to nanopriming, but a generalized hormetic curve should be treated as a hypothesis rather than a universal law. Beneficial and toxic thresholds depend on nanoparticle composition, primary and hydrodynamic size, morphology, surface functionalization, dissolution rate, exposure duration, crop genotype, developmental stage, and surrounding medium. A material may be beneficial in one crop and inhibitory in another at the same nominal concentration. Thus, each nanopriming formulation should establish an empirical response surface rather than assume that a single concentration range defines the ‘stimulatory zone’. **Figure 2** is therefore a conceptual framework, not a universal quantitative threshold (Singh et al., 2024; Zhao et al., 2024).

**Figure 2:**
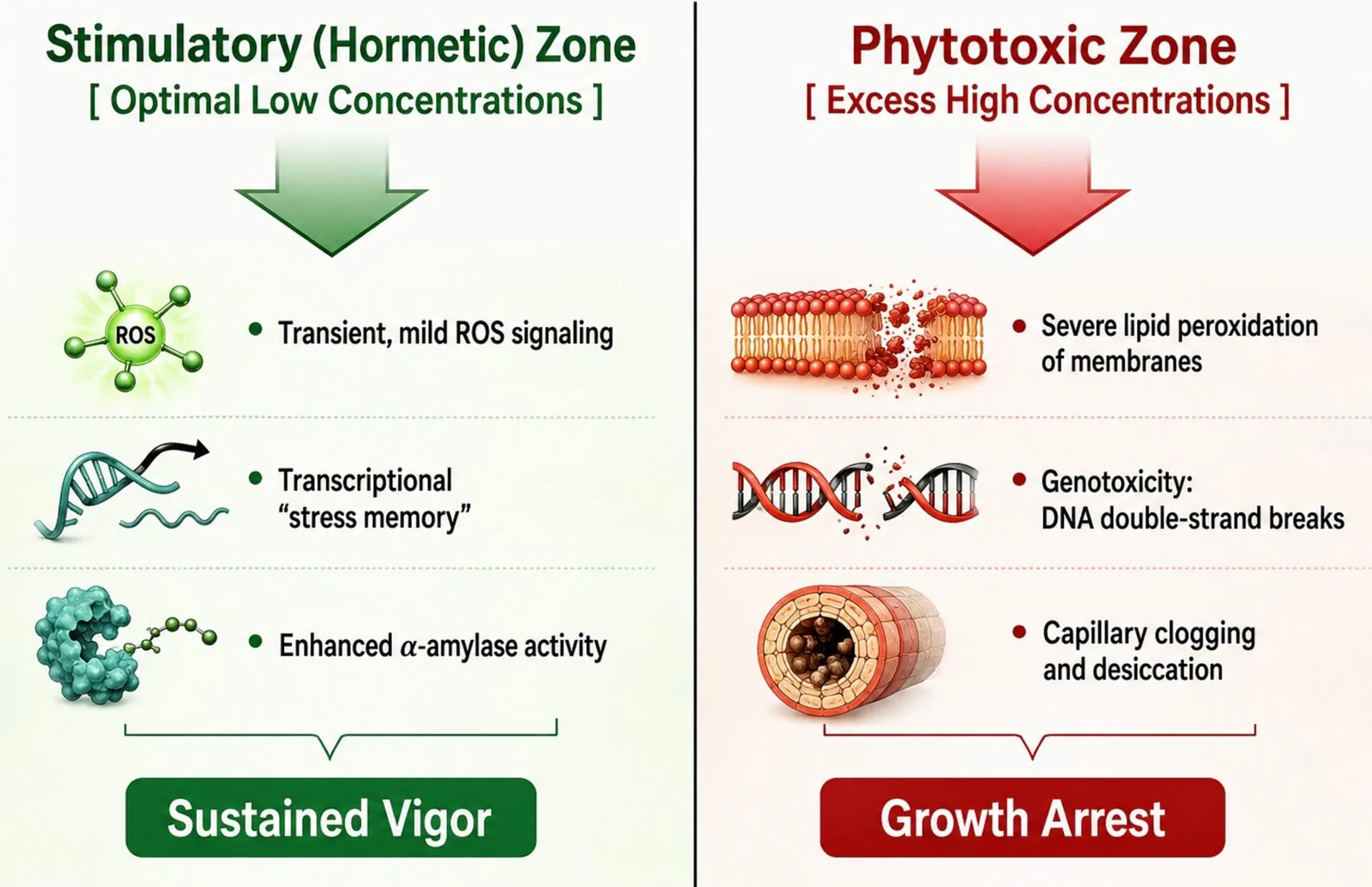
Biphasic dose response of nanoparticles in plants.

Phytotoxicity can arise through several non-exclusive mechanisms: persistent ROS generation, metal-ion release, membrane perturbation, DNA damage, impaired mitochondrial or chloroplast function, root-surface occlusion, altered nutrient balance, or toxicity of residual surfactants/capping agents. Exposure duration is equally important because a short priming pulse may be tolerated whereas repeated or chronic exposure can shift the same formulation into a damage state. Safety assessment should therefore report particle-size distributions, dissolution, surface chemistry, actual rather than nominal dose, exposure time, recovery period, and endpoints spanning germination, membrane integrity, oxidative markers, root architecture, microbiome effects, and yield.

Safer-by-design strategies should prioritize biocompatible ligands, controlled dissolution, minimized residual reagents, reproducible particle size, and application-specific dose windows. Biological or plant-mediated synthesis can reduce some hazardous reagents, but it does not automatically guarantee low toxicity: synthesis conditions, residual biomolecules, defect density, size, aggregation, and dose can all change environmental behavior. Green synthesis should therefore be treated as a process attribute that requires independent toxicological and environmental validation, not as a proxy for safety (Zhao et al., 2024; Atanda et al., 2025).

## 9 Transient nanocarrier delivery for transgene-free plant genome engineering

Smart nanocarriers used for genome editing extend the same design principles discussed for seed nanopriming—size, surface chemistry, cytosolic access, cargo protection, and stimulus-responsive release—into precision genetic interventions. The connection is translational rather than synonymous: most demonstrations of nanocarrier-mediated CRISPR delivery have used mature tissues, vacuum infiltration, foliar or other delivery routes rather than routine commercial seed priming. Their relevance to nano-enabled seed treatment lies in the prospect of combining transient cargo delivery with precision establishment or seedling-stage trait engineering.

Cas9/sgRNA ribonucleoprotein (RNP) delivery is distinct from stable transformation because the protein and guide RNA are transient cargos that can be degraded after editing, whereas plasmid DNA or stable vector integration may persist. A ‘transgene-free’ edited plant therefore refers to the absence of integrated foreign DNA in the final regenerated plant; it does not mean that no exogenous material was ever delivered. Regulatory classification is jurisdiction-dependent and should not be equated automatically with either conventional GMO status or universal exemption. In **Table 2**, transient cargo delivery and stable transformation are explicitly separated (Aslam 2023; Pausch et al., 2020).

**Table 2:** Nanocarrier platforms for transient genetic cargo delivery, with explicit separation of transgene-free editing from stable transformation.

Delivery platform	Cargo	Delivery mode	Immediate outcome	Stable transformation	Plant target/context	Relation to seed nanopriming
Arginine-functionalized nanohydroxyapatite	Cas9 RNP; plasmid DNA in some studies	Vacuum infiltration/passive transport	Transient RNP delivery can enable genome editing	RNP: no; plasmid: potentially yes	Embryos and mature tissues in proof-of-concept studies	Same size/charge/cargo-protection principles; not yet routine seed priming
SPION-based/green iron nanoparticles	Cas9 RNP or plasmid reporters	Magnetically assisted delivery	Cargo delivery followed by editing or reporter expression	Depends on cargo; RNP is transient	Embryonic/germline contexts in experimental systems	Potential future precision seed/embryo delivery
Star nanopolymers	siRNA and agrochemical cargos	Thermoresponsive or foliar/vacuum delivery	Controlled release of transient cargo	No by itself	Tomato leaf/root systems	Conceptually relevant to stimuli-responsive priming
Small ZIF-8	dsRNA/CRISPR cargo in reported systems	Acid-responsive degradation	Cargo release as framework destabilizes	Depends on cargo; RNP/dsRNA transient	Tobacco and Arabidopsis experimental systems	Model for triggered release rather than proof of seed transformation

## 10 Empirical review of nano-priming outcomes across crop families

The biological outcomes of seed nanopriming represent a complex interplay between nanomaterial design, core/hydrodynamic size, concentration, and crop-specific physiological thresholds (**Table 3**).

**Table 3:** Nano-priming outcomes across crop families, emphasizing the diversity of material, exposure, and evidence contexts.

Crop family	Crop species	Nanoparticle type & surface chemistry	Particle size (nm)	Applied concentration	Exposure duration	Physiological, biochemical & molecular outcomes	References
Fabaceae	Alfalfa (*Medicago sativa*)	Cerium Oxide Nanoparticles (CeO2NPs)	Not specified	50 mg/L	12 hours	Alleviates salinity (200 mM NaCl); germination 10%→62.7%; decreases ABA and JA; enhances α-amylase	Gao et. al., 2024
Poaceae	Maize (*Zea mays*)	Iron Oxide Nanoparticles (Fe2O3)	Not specified	75 mg/L	12 hours	Mitigates drought; RWC + 13%, WUE + 64%; elevates chlorophyll, flavonoids, ascorbic acid; reduces H2O2 and MDA	Mazhar et al., 2024
Poaceae	Maize (*Zea mays*)	ZnO NPs + 24-Epibrassinolide (EBL)	Commercial dry powder	100 mg/L ZnO + 0.2 μM EBL	Not specified	Reduces Na+ and Na+/K+ ratio under salinity; maximizes photosynthesis and Zn accumulation	Ahmad et al., 2023
Poaceae	Barley (*Hordeum vulgare*)	Zinc Oxide (ZnO) Nanoparticles	Not specified	Not specified	Not specified	Enhances salt tolerance; upregulates *CAT1* (79x), *NHX3* (208x), *SOS1*, *SOS3*; maintains K+/Na+	Abozaid et al., 2026
Poaceae	Barley	Nano-CeO2 vs Nano-TiO2	Not specified	150 mg/L CeO2/500 mg/L TiO2	Not specified	CeO2 targets roots (lowers ABA, raises GA3); TiO2 targets shoots (stimulates auxin, boosts photosynthesis)	Ye et al., 2026
Poaceae	Rice (*Oryza sativa*)	Zinc Oxide Nanoparticles (ZnONPs)	Not specified	25 ppm	24 hours	Ameliorates drought; increases height, chlorophyll, 1000-paddy weight; MDA -53%; proline and SOD/CAT/POD up	Mazhar et al., 2022b
Poaceae	Rice (*Oryza sativa*)	Chitosan Nanoparticles (CNPs)	50–100 nm SEM/373.5 nm DLS	50 μg/mL	Not specified	Alleviates salinity (0–250 mM NaCl); increases germination potential and antioxidant enzymes	Soni et al., 2023
Poaceae	Flax (*Linum usitatissimum*)	Iron Oxide Nanoparticles (Fe2O3)	Not specified	75 ppm	Not specified	Boosts water-stress resilience; stem length, dry weight, fiber yield up; MDA -66%, H2O2 -71%; SOD + 28%, CAT + 39%	Mazhar et al., 2022a
Poaceae	Wheat (*Triticum aestivum*)	Biogenic Manganese Oxide (MnO-NPs)	Not specified	80 mg/L	30 days pot trial	Mitigates wastewater stress; germination +57%, chlorophyll +53.5%; APX + 61%, CAT + 65%; electrolyte leakage -52%	Ishfaq et al., 2025
Solanaceae	Tomato (*Solanum lycopersicum*)	Selenium Nanoparticles (SeNPs)	Not specified	75 ppm	Field trials	Mitigates drought; MDA -28.9%, H2O2 -39.3%; upregulates ascorbate-glutathione cycle; ascorbic acid +26.4%	Ishtiaq et al., 2023
Solanaceae	Tomato	Auxin & Salicylic Acid Nanoformulations	Not specified	Not specified	Not specified	Polymeric carrier ensures controlled phytohormone delivery; mitigates terminal drought; preserves RWC	Mumithra Kamatchi et al., 2020
Cucurbitaceae	Watermelon (*Citrullus lanatus*)	Turmeric Oil Nanoemulsions & AgNPs	Not specified	Not specified	24 hours	Enhances germination of low-vigor triploid seeds; boosts glucose/fructose at 96h; yield +35.6% multi-location	Acharya et al., 2020
Fabaceae	Common Bean (*Phaseolus vulgaris*)	Citrate-coated Cobalt Ferrite (CoFe2O4)	Not specified	10–40 mg/L	6 hours	Safe developmental cycle; induces nitrogenase 343.3 μmol/h/plant; biofortifies progeny (K + 16%, Zn +27%)	Perea-Vélez et al., 2024

## 11 Future perspectives and research directions

The application of nanotechnology in seed priming has advanced considerably over the past decade, evolving from proof-of-concept studies to increasingly sophisticated delivery platforms capable of regulating multiple physiological and molecular processes simultaneously. Despite this progress, several scientific and technological challenges continue to limit large-scale agricultural implementation. Addressing these limitations will require interdisciplinary collaboration spanning plant biology, nanotechnology, materials science, computational modeling, environmental toxicology, and precision agriculture.

### 11.1 Development of next-generation smart nanocarriers

Current nanopriming systems have demonstrated substantial improvements in seed germination, stress tolerance, and nutrient-use efficiency. Nevertheless, many existing formulations respond to only a single environmental stimulus, restricting their adaptability under naturally fluctuating field conditions. Future nanocarriers should therefore incorporate multi-responsive delivery systems capable of simultaneously sensing changes in pH, temperature, redox potential, moisture availability, and specific biochemical signals associated with plant stress (Li et al., 2024).

### 11.2 Artificial intelligence and machine learning in nanopriming design

AI/ML can contribute to nanopriming only when tied to measurable physicochemical and biological endpoints. Graph neural networks (GNNs) can encode nanoparticle composition, ligand connectivity, particle topology, and surface descriptors to predict interactions across material libraries; Nano-QSAR models can relate composition, size, charge, hydrophobicity, dissolution, and exposure conditions to toxicity or efficacy; and molecular-dynamics simulations can estimate adsorption energies and contact configurations between functionalized nanoparticle surfaces and model pectin or lipid bilayers. For smart carriers, coarse-grained or atomistic simulations can further evaluate how pH-, ionic-strength-, and redox-dependent changes modify ligand protonation, pore gating, framework stability, or cargo release. A practical workflow is therefore: experimentally characterize a library of formulations → encode material and exposure descriptors → train interpretable Nano-QSAR/GNN models → validate predicted binding, uptake or toxicity experimentally → use molecular dynamics to interrogate the highest-value interfacial mechanisms → iteratively optimize the formulation. Such models should be trained on application-relevant media because a binding prediction in pure water may not describe behavior in seed exudate, apoplast, or soil pore water (Shelar et al., 2022).

### 11.3 Integration with multi-omics technologies

Although numerous studies have documented physiological improvements following nanopriming, the underlying molecular mechanisms remain only partially understood. Future investigations should integrate genomics, transcriptomics, proteomics, metabolomics, ionomics, and epigenomics to construct comprehensive regulatory networks describing nanoparticle–plant interactions. Multi-omics approaches will enable researchers to identify novel signaling pathways, characterize stress-responsive regulatory networks, and determine how different nanomaterials influence cellular metabolism across developmental stages.

### 11.4 Precision agriculture and digital farming

Future agricultural production systems will increasingly combine nanotechnology with digital monitoring platforms. Nanosensors capable of continuously detecting changes in soil moisture, nutrient availability, pathogen activity, or plant physiological status could provide real-time information for precision management of crop production. Integration of these sensing technologies with drone-based imaging, satellite remote sensing, Internet of Things (IoT) networks, and automated irrigation systems would allow site-specific application of nano-enabled formulations.

### 11.5 Green nanotechnology and sustainable manufacturing

Green synthesis should be framed as a route toward potentially lower reagent burden and improved circularity, not as evidence of intrinsic safety. Plant extracts, microorganisms, fungi, algae, and biodegradable polymers can act as reducing or capping agents, but their composition may vary with source, season, extraction conditions, and processing. These differences can change particle size, defect density, zeta potential, dissolution, and residual organic matter. For agricultural deployment, green routes should therefore be evaluated against conventional synthesis using the same criteria: batch reproducibility, purity, residual reactants, environmental transformation, phytotoxicity, microbial effects, and life-cycle cost.

### 11.6 Translational viability: cost, reproducibility, scale and regulation

The most realistic path to field translation is unlikely to be the immediate adoption of the most structurally complex nanocarrier. Biodegradable polymers such as chitosan may offer lower manufacturing complexity and compatibility with existing seed-coating workflows, whereas advanced MOFs and multifunctional core–shell systems can offer more precise release but may face higher synthesis cost, batch-to-batch variability, regulatory scrutiny, and uncertain soil transformation. Industrial seed treatment also requires compatibility with drying, storage, mechanical handling, dust control, coating adhesion, and germination standards. Reproducibility should therefore be demonstrated across seed lots, soil types, water chemistry, storage conditions, and multiple locations. Economic analyses should compare cost per hectare or seed rather than cost per laboratory dose, and regulatory dossiers should address worker exposure, food-chain residues, soil persistence, microbial effects, and transformation products (Athanasiou et al., 2025).

### 11.7 Long-term biosafety and regulatory harmonization

Despite encouraging experimental results, comprehensive evaluation of long-term environmental and food safety remains essential before nano-enabled seed priming can be widely adopted. Most current investigations emphasize short-term physiological responses, whereas relatively few studies have examined chronic environmental exposure, multigenerational effects, or nanoparticle behavior across complete agricultural ecosystems. Future research should therefore include long-term field trials evaluating nanoparticle persistence, environmental transformation, trophic transfer, soil microbial dynamics, and potential bioaccumulation within food chains.

### 11.8 Bridging the gap between laboratory research and field application

A persistent limitation of existing nanopriming research is the predominance of controlled laboratory and greenhouse experiments. Environmental variability under field conditions—including fluctuations in temperature, rainfall, soil composition, microbial diversity, and management practices—may substantially influence nanoparticle performance. Future investigations should prioritize multi-location field experiments involving diverse crop species, soil types, and climatic regions. Such studies will provide more realistic assessments of agronomic performance, economic viability, and environmental safety.

## 12 Conclusion

Nanopriming is best understood as a materials–plant interaction problem rather than a single agronomic technology. The literature supports beneficial effects across many crops and stress contexts, but the magnitude and direction of response depend on formulation-specific physicochemical properties, dose, exposure duration, crop genotype, and environmental matrix. The synthesis highlights four persistent uncertainties: how interfacial properties translate into tissue-level uptake; which ROS/Ca2+/MAPK/hormone pathways are directly triggered by individual nanomaterials; how soil transformations and eco-coronas alter bioavailability; and whether laboratory dose windows remain effective and safe under field conditions.

The immediate priorities are therefore mechanistic and translational. Studies should report standardized particle and dispersion descriptors, distinguish intact-particle from ion/cargo effects, use factorial designs for combined treatments, establish material- and crop-specific dose–response surfaces, and validate mechanisms with spatially resolved imaging and multi-omics. For commercial deployment, biodegradable and reproducible formulations compatible with existing seed-coating infrastructure may offer the most realistic near-term route, while complex MOFs and hybrid systems should be advanced where their added functionality clearly offsets their manufacturing and regulatory burden. Long-term environmental transformation, soil microbiome effects, food-chain exposure, storage stability, and multi-location field performance must become standard evaluation endpoints. Finally, transgene-free nanocarrier-enabled editing and AI-guided formulation design should be framed as emerging precision tools rather than established extensions of routine seed priming.

Overall, the major research gap is not whether nanomaterials can influence plant performance, but how to predict when, for which crop, at what dose, and under which environmental conditions a specific nanoformulation will deliver a reproducible benefit without unacceptable ecological or food-safety trade-offs. Addressing that predictive gap is the central requirement for responsible field deployment.
